# Evaluation of Washing with Sodium Hypochlorite, Ultraviolet Irradiation, and Storage Temperature on Shell Egg Quality During Storage

**DOI:** 10.3390/foods14132156

**Published:** 2025-06-20

**Authors:** Hui-Chuan Yu, I-Chi Chen, Fa-Jui Tan

**Affiliations:** Department of Animal Science, National Chung Hsing University, Taichung 402, Taiwan; cathyyu55@gmail.com (H.-C.Y.); loves6522@gmail.com (I.-C.C.)

**Keywords:** eggshell decontamination, ultraviolet treatment, sodium hypochlorite, storage stability, microbial safety

## Abstract

Shell eggs are susceptible to fecal contamination, facilitating the adhesion of microorganisms to the eggshell surface. The consumption of such eggs, especially when unwashed or raw, poses potential health risks to consumers. This study aimed to evaluate the effects of unwashed control, sodium hypochlorite (NaOCl) sanitization (150 ppm), and the combination of NaOCl and ultraviolet (UV) sanitization on the quality of eggs stored at varying temperatures over a four-week period. The findings demonstrated that NaOCl sanitization alone reduced surface bacterial counts by 1.23 log_10_ CFU/mL, while the combination of NaOCl and UV-C irradiation achieved a greater reduction of 1.48 log_10_ CFU/mL compared to the unwashed group. After two weeks of storage, unwashed egg groups (UC and UR) exhibited higher eggshell strength compared to NaOCl-sanitized groups (*p* < 0.05); however, this did not significantly influence internal contamination. Prolonged storage, particularly under refrigeration, led to increased hydroxyl (OH) group peak intensities on the eggshell, indicating dehydration and the formation of fissures in the cuticle. Elevated storage temperatures and extended durations adversely affected egg quality, whereas UV treatment did not have a detrimental impact. In conclusion, to ensure the safety and quality of shell eggs, it is recommended that they undergo NaOCl sanitization, UV irradiation, and be stored under refrigerated conditions.

## 1. Introduction

Eggs are widely recognized as a nutrient-dense food, offering essential components vital for human health [1]. They serve as an excellent source of high-quality protein, characterized by high digestibility (98%) and biological value (94%) due to their favorable essential amino acid composition [1]. Additionally, eggs are rich in vitamins and minerals, notably selenium, riboflavin, vitamin B12, and biotin. Beyond their basic nutritional value, eggs have been associated with various health benefits [2]. For example, daily consumption of three eggs as part of a carbohydrate-restricted diet has been linked to increased satiety [3], elevated high-density lipoprotein (HDL) levels, and reductions in markers of metabolic syndrome [4]. Although earlier studies raised concerns about dietary cholesterol, recent findings suggest that consuming more than six eggs per week does not increase the risk of stroke in healthy individuals [5]. These insights position eggs as a valuable dietary component with broad health-promoting potential [1].

The eggshell plays a critical role in protecting the egg’s internal contents by serving as the primary physical barrier against microbial invasion [6]. Its multilayered structure consists of the outer cuticle, pores, and inner and outer shell membranes, forming a comprehensive defense structure [7]. Microbial contamination of the eggshell typically arises from horizontal transmission after laying, through exposure to fecal matter, dust, and contaminated surfaces [8]. Additionally, vertical transmission from microorganisms present in the hen’s reproductive tract may lead to contamination before the egg is laid [1,8]. Pathogens such as *Listeria monocytogenes* and *Salmonella* Typhimurium are frequently found on eggshells. *L. monocytogenes* can form biofilms on the eggshell surface [7], whereas *S.* Typhimurium has the ability to penetrate the shell, a process influenced by its ultrastructural characteristics [9]. Consumer perception studies indicate widespread concern regarding microbial risks in eggs, particularly involving *Salmonella*, *Listeria*, and *Escherichia coli*. Therefore, the implementation of effective hygiene control measures is crucial to ensuring the safety of shell eggs for consumption [6].

Commercial egg washing typically involves four key stages: wetting, sodium hypochlorite (NaOCl) sanitization, rinsing, and drying [10]. Disinfectants used in the sanitization stage effectively reduce microbial loads on the eggshell surface. However, the washing process, equipment type, and handling practices may impact the cuticle’s integrity [11,12]. Some studies have shown that washing can reduce cuticle thickness, potentially compromising egg quality and shelf life, especially under suboptimal storage conditions [13]. In contrast, other research suggests that washing does not significantly alter cuticle quality, depending on the process used [11].

Among various eggshell disinfection technologies, NaOCl and ultraviolet (UV) irradiation are frequently employed or studied due to their complementary strengths and effectiveness. NaOCl is a well-established disinfectant in the food industry, known for its efficacy against a wide range of pathogens by disrupting bacterial cell walls and denaturing proteins [7,14]. In Taiwan, NaOCl is approved by the Ministry of Health and Welfare as a food-grade sanitizer for cleaning food, utensils, containers, and packaging [15]. Although NaOCl may cause some discoloration, studies show that combining it with UV-C irradiation mitigates this effect while enhancing disinfection efficacy [7]. UV-C is a non-thermal method that inactivates microorganisms by damaging their DNA, with the advantage of leaving no chemical residues and preserving the sensory qualities of food products [2,16]. UV irradiation has demonstrated effectiveness in reducing surface bacteria such as *Salmonella* without significantly affecting eggshell appearance or internal egg quality [7,13,17]. Furthermore, UV-C is cost-effective and suitable for high-throughput egg processing. Combining UV-C with NaOCl has shown a synergistic effect in reducing biofilms and microbial loads while minimizing quality degradation. Therefore, this combination presents a balanced approach between microbial safety and quality preservation, making it a promising method for use in Taiwan’s egg production industry.

In Taiwan, eggs are primarily marketed as bulk eggs (unwashed and ungraded), Certified Agricultural Standards (CAS) eggs, or Traceable Agricultural Products (TAP) eggs [18,19]. Among these, bulk eggs dominate the market and are collected directly from henhouses for sale without undergoing washing or grading processes. Over 80% of layer hens in Taiwan are reared in open housing systems, where eggs frequently come into contact with manure, litter, and dust, increasing the risk of microbial contamination. Compounding this risk, eggs are often distributed and stored without proper sanitation or cold-chain systems [20,21]. Consequently, concerns about the microbial safety and shelf life of shell eggs are particularly pronounced in Taiwan.

Although the Taiwanese government has been promoting egg washing and grading practices for years, the adoption rate remains relatively low. As of May 2025, only approximately 37% of shell eggs are processed through washing and grading systems [22]. This cautious adoption mirrors the European Union’s perspective, where egg washing is restricted due to concerns over cuticle degradation and subsequent bacterial penetration [23,24]. In contrast, the United States and Canada adopt a cleaning-centered model in which eggs are thoroughly washed and refrigerated (typically below 7 °C), a policy shaped by consumer preferences and retail expectations [25,26]. While the U.S. model improves surface hygiene, critics argue it may increase moisture loss and permeability due to cuticle damage [11,13]. Conversely, the EU model prioritizes cuticle preservation, though it tolerates visible fecal contamination and allows room-temperature storage with temperature fluctuations [13]. However, research suggests that the U.S. and Canadian approach may be more effective in controlling pathogen survival and maintaining internal quality over prolonged home storage—an increasingly relevant issue given rising egg prices and extended household storage durations [25].

Taiwan’s egg industry currently aligns more closely with the European model, favoring unwashed eggs due to concerns over cuticle damage. However, in response to global warming and rising ambient temperatures, the Taiwanese government increasingly supports hygienic washing practices to mitigate food safety risks associated with microbial growth on eggshells [20,21,22]. These diverging approaches—between an EU-like market preference and a U.S.-like regulatory direction—highlight the urgency of reconciling food safety with egg quality.

Given this context, research that evaluates post-washing interventions such as NaOCl and UV-C irradiation is critical to guiding policy and addressing the industry’s hesitation toward washing practices. Our findings may help reduce concerns among Taiwanese egg producers regarding internal contamination and quality loss in washed eggs. Therefore, the objectives of this study were to: (1) evaluate the effects of unwashed control, NaOCl sanitization, and the combination of NaOCl and UV sanitization on the quality of eggs stored at different temperatures for four weeks; and (2) assess the antimicrobial efficacy of NaOCl alone versus its combination with UV irradiation in reducing microbial loads on the eggshell surface. The findings aimed to provide scientific evidence supporting effective decontamination approaches for improving the microbial safety and quality of shell eggs in the Taiwanese market.

## 2. Materials and Methods

### 2.1. Egg Samples

A total of 600 fresh and visibly clean eggs were collected from the same production flock. The external appearance of the eggs was assessed based on the “Guidelines for Washing and Grading of Fresh Eggs” issued by the Ministry of Health and Welfare, Taiwan. According to these guidelines, accepted washed eggs must exhibit clean and intact shells, free from foreign substances, significant stains, or abnormal discoloration. Eggs with excessive shell fragility, soft shells, cracks, fractures, or obvious contamination with feces or severe dirt were excluded.

The eggs were divided into three treatment types: unwashed control, NaOCl sanitization, and the combination of NaOCl and UV sanitization. Each treatment type included two storage temperature conditions: 7 °C (refrigerated) and 25 °C (room temperature). Specifically, 200 unwashed eggs were allocated to the unwashed control group and stored at either 7 °C (UC group) or 25 °C (UR group). Another 200 eggs were sanitized using NaOCl solution and divided into two groups: 7 °C (NC group) and 25 °C (NR group). The remaining 200 eggs were treated with NaOCl solution followed by UV radiation and stored at 7 °C (NUVC group) and 25 °C (NUVR group). All eggs were stored for 4 weeks before subsequent experimental analysis.

### 2.2. Washing Process

A total of 400 eggs designated for the treatment groups were processed at a local egg packing station using a commercial egg-washing procedure commonly practiced in Taiwan. The eggs were placed on a conveyor belt and cleaned using a commercial egg washer (Oracion6000, Nabel, Kyoto Japan), which combined mechanical brushing (30 s) with high-pressure water spraying. The wash water contained 150 ppm NaOCl and was maintained at 39 °C. According to previous studies, increasing the NaOCl concentration beyond 150 ppm does not significantly enhance the antibacterial effect against *L. monocytogenes* [7]. Therefore, 150 ppm was selected as the benchmark for evaluating both individual and synergistic effects, and this concentration also complies with Taiwan’s food safety regulations, which allow a maximum of 200 ppm for food-contact sanitizing agents.

Among the NaOCl-treated eggs, 200 were assigned to the “NaOCl sanitization group”, while the remaining 200 underwent an additional UV-C irradiation step at a wavelength of 253.7 nm, using a commercial UV system (SP104ND, Nabel, Kyoto, Japan). During the UV-C treatment, eggs rotated and moved along the conveyor belt with the aid of rollers, ensuring that the entire surface of each egg received full and even UV-C exposure. The exposure time was set to 10 s based on the calculated belt speed and system configuration. Eggs that did not receive any sanitization treatment were designated as the “unwashed control group”. All treated eggs were subsequently packaged and used for further experimental analysis.

### 2.3. Microbial Analysis of Eggshell Surface and Egg Internal Contents

The primary objective of this microbial analysis was to quantify mesophilic aerobic bacteria, expressed as total aerobic plate count (TAPC), on the eggshell surface and in internal contents after different sanitization treatments. This indicator was selected to provide a general assessment of microbial contamination and to evaluate the efficacy of washing and disinfection interventions.

For eggshell surface analysis, TAPC were determined following the procedures outlined by the Taiwan Food and Drug Administration (TFDA) [27] and Musgrove et al. [28]. Each egg was placed into a sterile plastic bag containing 10 mL of 0.1% peptone diluent (buffered peptone water, BPW; Difco, Tucker, GA, USA) and gently massaged for 1 min. The resulting rinse solution was serially diluted in BPW and plated in triplicate onto plate count agar (Difco, Taipei, Taiwan). Plates were incubated at 37 °C for 48 h, and colony counts were recorded as colony-forming units per milliliter (CFU/mL).

For internal content analysis, the microbial enumeration followed the protocols provided by the TFDA [27] and Jones et al. [25]. The eggshells were externally disinfected with 75% ethanol and aseptically cracked in a laminar flow hood. The contents were transferred into sterile stomacher bags and homogenized with a paddle blender (BagMixer, InterScience, Saint-Nom-la-Breteche, France) for 30 s. Homogenates were serially diluted in BPW and plated onto plate count agar, followed by incubation at 37 °C for 48 h. Colony counts were expressed as CFU/g.

The same batch of eggs was used for both surface and internal microbial analyses to ensure consistency in sample origin and treatment conditions. Eggs from the control group (unwashed), NaOCl sanitization group, and NaOCl + UV-C sanitization group were analyzed to compare the reduction in microbial loads among treatments.

$$Total\;aerobic\;plate\;count\;(CFU/g\;or\;CFU/mL)\;=\;[(\frac{Aa+Ab}{2})\times A+(\frac{Ba+Bb}{2})\times B]\times \frac{1}{2}$$

where:

Aa, Ab: colony counts on duplicate plates at dilution level A
Ba, Bb: colony counts on duplicate plates at dilution level B
A, B: corresponding dilution factors.

The final results were converted to logarithmic values (log_10_ CFU/g or CFU/mL) for statistical analysis and reporting.

### 2.4. Measurement of Eggshell Quality

Air cell size was measured using a micrometer [29]. Eggshell strength was assessed using a tensile and compression strength tester (HT-8116, Hung-Ta, Taichung, Taiwan), expressed in kg/cm^2^. Eggshell thickness at the top, middle, and bottom was measured after removing the shell membrane, using a thickness gauge (FHK FN-595, Ozaki, Osaka, Japan) [30]. These measurements were conducted on eggs from the control group (unwashed), NaOCl sanitization group, and NaOCl + UV sanitization group to evaluate the structural impact of each treatment.

For hydroxyl (OH) content analysis, a 0.5 × 0.5 cm eggshell piece was analyzed using attenuated total reflectance–Fourier transform infrared (ATR–FTIR) spectroscopy (JASCO 6200, Tokyo, Japan), following the method of Rodríguez-Navarro et al. [31] and Liu et al. [13]. Spectra were recorded at 2 cm^−1^ resolution over 100 scans. The intensity of the OH absorption peaks was used to assess water content and dehydration levels in the eggshell cuticle across the three treatment groups.

### 2.5. Ultrastructural Assessment

Scanning electron microscopy (SEM) was used to examine the cuticle ultrastructure, with procedures adapted from Mahato et al. [32]. A 0.5 cm^2^ eggshell section was cut from the equator of the egg, rinsed gently with distilled water to remove surface debris, and allowed to air-dry. The dried shell samples were mounted on stubs, sputter-coated with gold–palladium (JFC-1600, JEOL, Tokyo, Japan), and observed under a SEM (JSM-6700F, JEOL, Japan) at 1000× magnification.

### 2.6. Measurement of Egg Internal Quality

Egg internal quality was evaluated by measuring Haugh unit (HU), yolk index (YI), thick albumen ratio, pH, and moisture content. Each egg was cracked and the contents were gently poured onto an egg quality stand (FHK NFN-381, Ozaki, Japan). The thick albumen and yolk heights were measured with a quality gauge (FHK NFR3, Ozaki, Japan). HU was calculated using the equation:

HU = 100 × log (H − 1.7w^0.37^ + 7.6)

where

H is albumen height (mm) and w is egg weight (g) [13].
YI was determined as yolk height/yolk width [33].

To calculate the thick albumen ratio, albumen was passed through a 2 mm nylon mesh sieve. The volumes of thick (residue) and thin (filtrate) albumen were recorded to compute the ratio [34]. The albumen and yolk were homogenized separately (BagMixer, InterScience, France) for 20 s, and the pH was measured using a digital pH meter (PHM 210, Radiometer, France) [35]. The moisture content was determined according to AOAC official methods [36].

### 2.7. Statistical Analysis

This study employed a split-plot design, with treatment groups serving as whole plots and storage weeks (0, 1, 2, 3, and 4) as sub-plots, to evaluate egg quality changes during storage. Independent sampling was conducted for microbiological analysis, air cell size, eggshell strength, hydroxyl content, albumen and yolk quality, and SEM assessment. The minimum sample size per group was approximately 85 eggs. To accommodate potential sample loss, contamination, and additional testing requirements, a 20% reserve was included, ensuring that each treatment group remained under 100 eggs. In total, 600 eggs were utilized to maintain the reliability and robustness of the experimental results. Data were analyzed by one-way analysis of variance (ANOVA), accounting for both whole plot and sub-plot effects inherent in the split-plot design. Tukey’s multiple comparison test was applied to assess statistical significance at *p* < 0.05. All statistical analyses were performed using SAS software (Version 9.4; SAS Institute Inc., Cary, NC, USA).

## 3. Results and Discussion

### 3.1. Effect of Bacteria Survival on Eggs with Different Treatments During Storage

Table 1 presents the bacterial counts on eggshells and internal contents of eggs subjected to different treatments and stored at either 7 °C or 25 °C for four weeks. The unwashed control group (U) exhibited significantly higher microbial loads on the eggshell surface than both the NaOCl-treated group (N) and the NaOCl + UV-C irradiation group (NUV) (*p* < 0.05). Specifically, NaOCl treatment alone reduced surface bacterial counts by 1.23 log_10_ CFU/mL, and the combined NaOCl + UV-C treatment achieved a reduction of 1.48 log_10_ CFU/mL, relative to the unwashed group.

These results confirm that both NaOCl and NaOCl + UV-C treatments are effective in reducing eggshell surface contamination, with the combined treatment showing the greatest efficacy. The antimicrobial activity of NaOCl is primarily due to the presence of hypochlorite ions, which elevate water pH and disrupt microbial cell functions [14]. In contrast, UV-C irradiation inactivates microorganisms by inducing DNA damage and disrupting cell membranes [14,16]. The superior performance of the combined treatment suggests a potential synergistic effect between chemical and physical sanitization mechanisms.

However, unlike non-porous surfaces, such as stainless steel—where synergistic effects of NaOCl and UV-C increase with higher doses—studies have shown that such synergy on eggshells tends to be weaker, inconsistent, and often independent of treatment intensity [7]. In some cases, high UV-C dosages may even exert antagonistic effects. This phenomenon is likely due to the rough and porous microstructure of eggshells, which allows microorganisms to reside in crevices that limit disinfectant access and reduce overall efficacy [7].

Compared to other methods, such as hydrogen peroxide and pulsed UV light, the NaOCl and NaOCl + UV-C treatments in this study achieved comparable microbial reductions (~1.23 to 1.48 log_10_ CFU/mL). While hydrogen peroxide has been shown to be effective in inactivating pathogens, its application may cause surface discoloration and raise concerns about residual safety [28,37]. Similarly, pulsed UV light offers rapid and non-thermal disinfection, but typically requires high-energy xenon-based systems, which increase operational cost and complexity, limiting its current adoption in regions like Taiwan [17,38]. Our results suggest that NaOCl + UV-C offers a practical balance between microbial efficacy and operational feasibility, making it a promising strategy for enhancing egg safety in subtropical production environments.

In contrast, the internal contents of the eggs remained largely unaffected by either the sanitization method or storage temperature. Across all treatment groups and storage conditions, TAPC in egg contents remained below 10 CFU/g throughout the 4-week storage period. This indicates that both NaOCl and NaOCl + UV-C treatments effectively reduce surface contamination without promoting bacterial penetration into the egg interior, thereby maintaining internal microbial safety during storage.

### 3.2. Eggshell Quality

**Figure 1:**
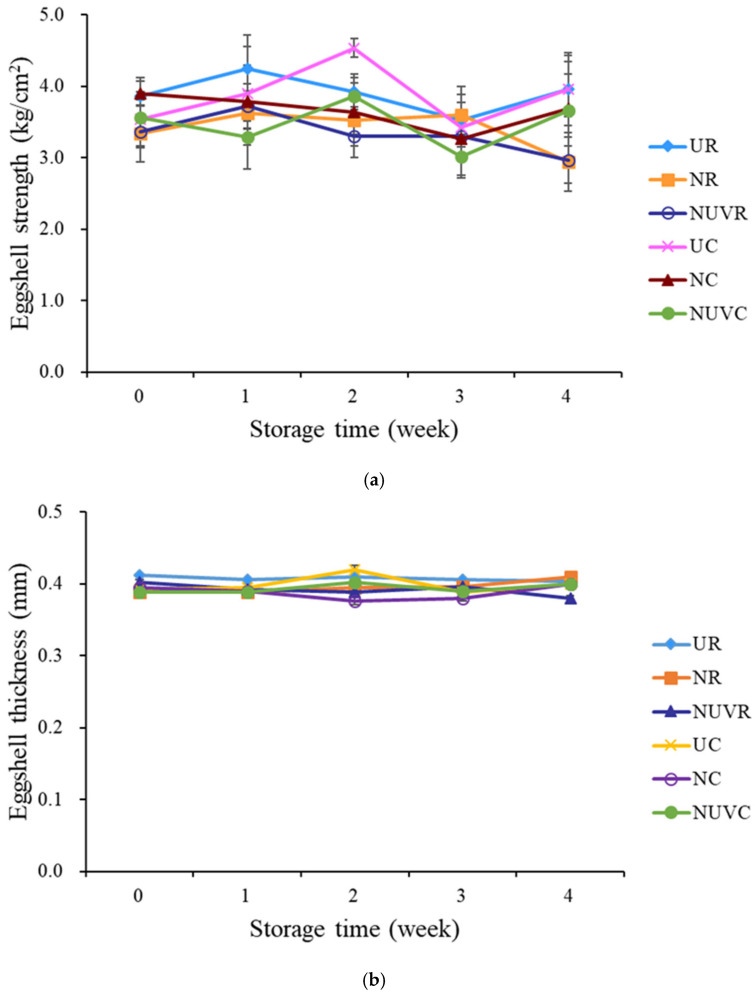
Change in (**a**) eggshell strength, and (**b**) eggshell thickness of eggs with different washing treatments and stored at 7 °C and 25 °C for 4 weeks. UR: unwashed and stored at 25 °C; NR: washed with NaOCl sanitizer and stored at 25 °C: NUVR: washed with NaOCl sanitizer, UV-irradiated and stored at 25 °C; UC: unwashed and stored at 7 °C; NC: washed with NaOCl sanitizer and stored at 7 °C; NUVC: washed with NaOCl sanitizer, UV-irradiated and stored at 7 °C.

Reduced eggshell strength is associated with a higher incidence of shell breakage, which leads to economic losses due to increased product rejection and microbial risk. As shown in Figure 1a, eggs in the unwashed control groups (UC and UR) exhibited significantly greater shell strength compared to those treated with NaOCl (NC and NR) and NaOCl + UV-C (NUVC and NUVR) after two weeks of storage (*p* < 0.05). This reduction in strength among washed eggs is likely due to mechanical abrasion during the brushing process, which may compromise the structural integrity of the shell. Chousalkar et al. [24] and Samiullah and Roberts [2] reported that such abrasion can partially or completely remove the cuticle layer, a critical barrier that not only contributes to shell strength but also enhances resistance to microbial penetration. Damage to the cuticle can lead to microstructural defects, such as exposed pores and surface microcracks, as observed in this study via SEM. These results are consistent with findings by Gole et al. [9], who demonstrated increased trans-shell bacterial penetration in mechanically washed eggs. Although direct quantitative data on strength reduction due to brushing are limited, the observed weakening in both the NaOCl and NaOCl + UV-C treatment groups highlights the vulnerability of the eggshell to physical manipulation. The relationship between shell strength and microbial contamination remains unclear. Jones and Musgrove [39] reported a weak correlation between eggshell strength and the presence of *Salmonella Enteritidis* on the shell surface or in internal contents. In our study, bacterial loads in internal contents remained low and did not differ significantly among unwashed, NaOCl-treated, and NaOCl + UV-treated groups (Table 1), suggesting that shell strength had a minimal impact on internal contamination during storage.

The extent of mechanical damage may vary depending on the washing system design. Leleu et al. [11] found that applying water and sanitizer as a spray—rather than direct contact brushing—can reduce shell surface damage. In addition, Keklik et al. [17] showed that pulsed UV light had no detrimental impact on shell strength, which aligns with our observation that the NaOCl + UV-C group did not show further reductions in shell strength compared to the NaOCl-only group.

As shown in Figure 1b, eggshell thickness did not significantly differ between treatment groups over the storage period (*p* > 0.05). This result is consistent with findings by Tilki and Saatci [40], who observed no significant change in eggshell thickness after 35 days of storage. While unwashed eggs initially appeared to have slightly thicker shells, the differences were not statistically significant after washing, indicating that neither NaOCl nor NaOCl + UV-C treatment meaningfully altered shell thickness.

**Figure 2:**
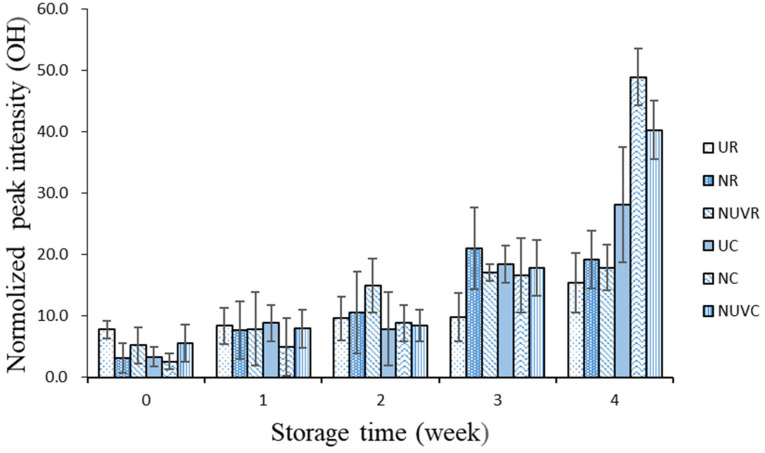
Change in OH band intensity of attenuated total reflection-Fourier transform infrared spectroscopy peaks associated with main chemical components of the eggshell cuticle with different washing treatments and stored at 7 °C and 25 °C for 4 weeks.

Figure 2 illustrates the temporal changes in ATR–FTIR spectral intensities related to the composition of the eggshell cuticle. The presence of OH peaks corresponds to the water content at the eggshell surface. In this study, OH peak intensity increased significantly with storage time (*p* < 0.05), and was more pronounced in eggs stored at 7 °C compared to 25 °C. According to Rodríguez-Navarro et al. [31], cuticle dehydration and fissure formation occur over time, exposing gas-exchange pores and facilitating water migration from the albumen to the eggshell surface. The intensified OH signal observed under refrigeration reflects this enhanced surface hydration due to moisture loss and vapor diffusion, particularly under lower temperature and moderate humidity (approximately 50%).

**Figure 3:**
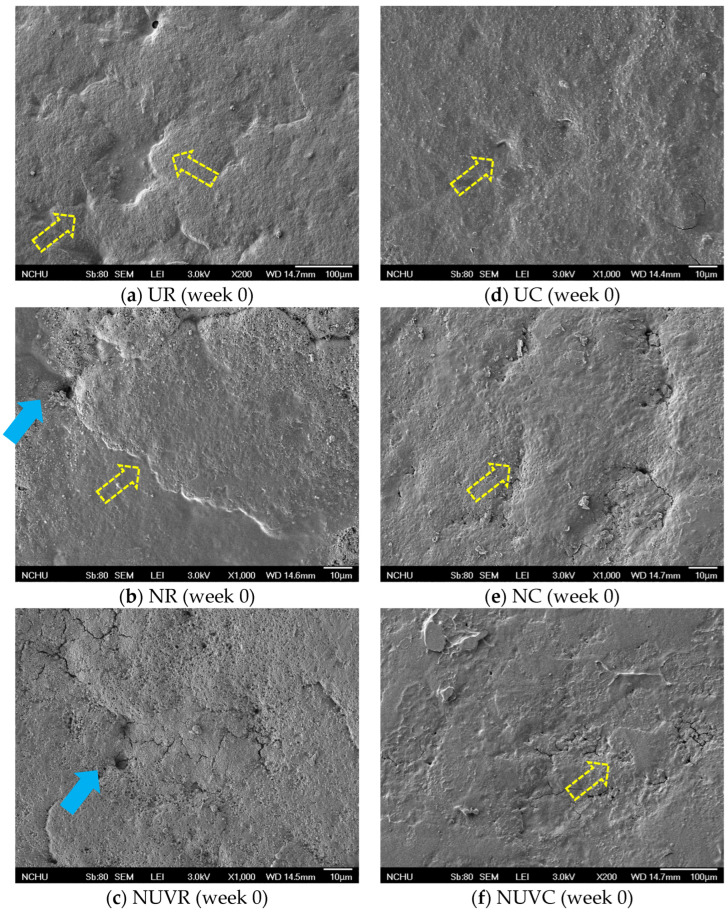

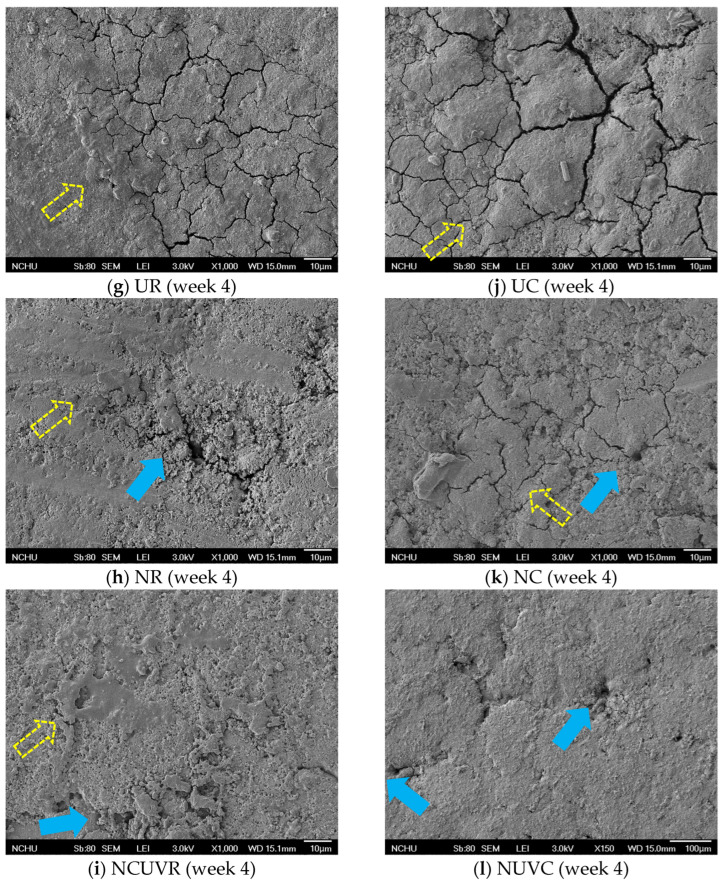
Scanning electron microscope (SEM) photographs of eggshell (top-view, ×1000). UR: unwashed and stored at 25 °C; NR: washed with NaOCl sanitizer and stored at 25 °C: NUVR: washed with NaOCl sanitizer, UV-irradiated, and stored at 25 °C; UC: unwashed and stored at 7 °C; NC: washed with NaOCl sanitizer and stored at 7 °C; NUVC: washed with NaOCl sanitizer, UV-irradiated, and stored at 7 °C. Pore is indicated by the solid arrow; cuticle is indicated by the hollow dashed line arrow.

Chousalkar et al. [24] reported that unwashed eggs typically retain a more intact cuticle layer compared to washed eggs. SEM analysis further confirmed the deterioration of the cuticle layer under different treatments. As shown in Figure 3a,d, unwashed eggs (UC and UR) retained a more intact, rough, and uneven cuticle morphology, with visible pore occlusion. The non-uniform texture is consistent with previous descriptions of natural cuticle coverage [2]. In contrast, eggs subjected to NaOCl (NC and NR) or NaOCl + UV-C (NUVC and NUVR) treatments displayed clear signs of cuticle erosion, including exposed pores, surface cracks, and residual debris (Figure 3b–f). These observations are consistent with studies indicating that disinfectants such as sodium carbonate, cetylpyridinium chloride, trisodium phosphate, and NaOCl can damage the cuticle by denaturing shell matrix proteins [1,2,9,10,14,41]. The cuticle contains hydrophobic protein structures that are sensitive to oxidative or alkaline agents [42], and Favier et al. [43] proposed that interaction with these agents may lead to protein denaturation and subsequent shedding of the cuticle.

Mechanical abrasion likely contributed further to cuticle loss, particularly in the brushing process applied during sanitization [11,13,44]. Despite the additional UV-C exposure, eggs in the NaOCl + UV-C groups (NUVC and NUVR) did not exhibit more extensive cuticle damage than those in the NaOCl-only groups, supporting previous findings that UV-C alone does not exacerbate structural damage [17] (Figure 3c,f).

Interestingly, after four weeks of storage, SEM images of unwashed eggs revealed visible surface cracks, particularly in those stored at 7 °C, suggesting that cold-induced dehydration may also weaken the cuticle. These results are in line with prior findings that prolonged refrigeration promotes the formation of a fissured cuticle network [31]. Additionally, larger pores were frequently observed in the washed and combined treatment groups, especially in the NaOCl + UV-C group (NUVC), indicating that combined chemical and physical sanitization, along with storage stress, may further compromise cuticle integrity [13].

### 3.3. Albumen Quality

**Figure 4:**
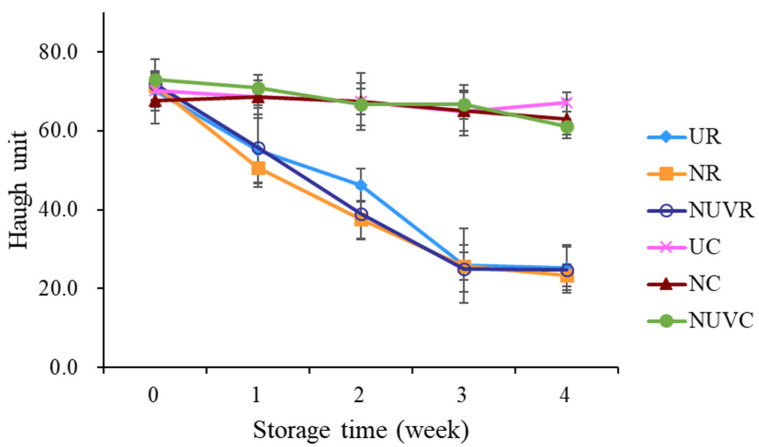
Change in Haugh unit of eggs with different washing treatments and stored at 7 °C and 25 °C for 4 weeks. UR: unwashed and stored at 25 °C; NR: washed with NaOCl sanitizer and stored at 25 °C: NUVR: washed with NaOCl sanitizer, UV-irradiated, and stored at 25 °C; UC: unwashed and stored at 7 °C; NC: washed with NaOCl sanitizer and stored at 7 °C; NUVC: washed with NaOCl sanitizer, UV-irradiated, and stored at 7 °C.

The HU is the widely accepted indicator of egg albumen quality and is used as a grading standard by the United States Department of Agriculture (USDA), which classifies eggs as Grade AA (>72), A (71–60), B (59–31), and C (<31) [45]. In this study, HU scores declined progressively with increasing storage time and temperature (Figure 4). Eggs stored at 25 °C (UR, NR, NUVR) exhibited a more rapid reduction in HU values over the 4-week period compared to those stored at 7 °C (UC, NC, NUVC).

After two weeks of storage at 25 °C, HU scores of eggs in the UR group remained slightly higher than those in the NR and NUVR groups, with average values of 25.86, 25.72, and 25.03, respectively. However, by the third week, all groups stored at 25 °C had HU values below 30, placing them in USDA Grade C or lower, and therefore no longer considered suitable for consumption.

**Figure 5:**
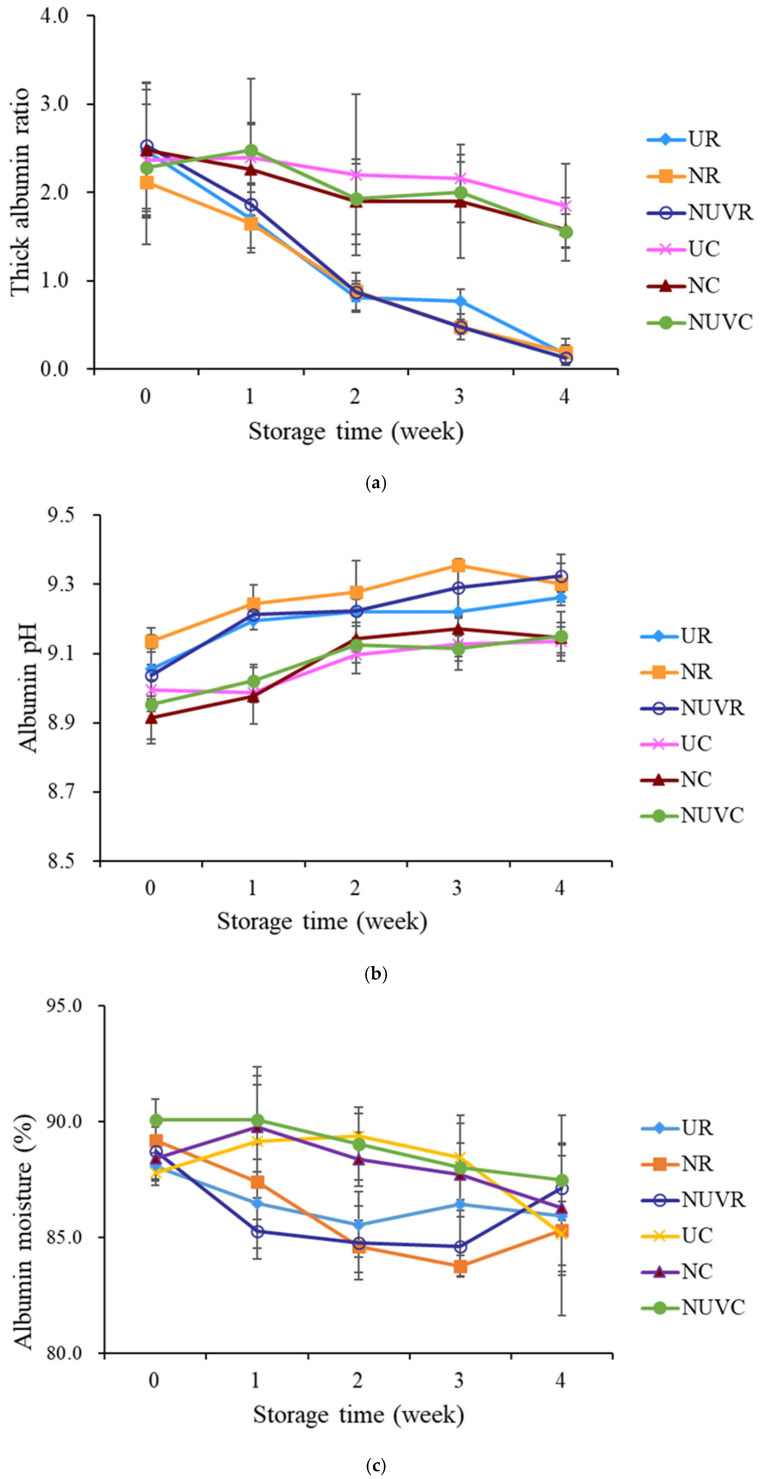
Change in (**a**) thick albumen ratio, (**b**) albumen pH value, and (**c**) albumen moisture content of eggs with different washing treatments and stored at 7 °C and 25 °C for 4 weeks. UR: unwashed and stored at 25 °C; NR: washed with NaOCl sanitizer and stored at 25 °C: NUVR: washed with NaOCl sanitizer, UV-irradiated, and stored at 25 °C; UC: unwashed and stored at 7 °C; NC: washed with NaOCl sanitizer and stored at 7 °C; NUVC: washed with NaOCl sanitizer, UV-irradiated, and stored at 7 °C.

In contrast, eggs stored at 7 °C maintained albumen quality within the Grade A range across all treatment groups (UC, NC, NUVC), with no significant differences observed among them. This finding underscores the critical role of cold storage in preserving internal egg quality, regardless of sanitization method. A similar trend was observed in the thick albumen ratio (Figure 5a), which declined more sharply at higher temperatures. These results are consistent with those reported by Jones et al. [25] and Liu et al. [13], who found that elevated temperatures accelerate albumen degradation. Moreover, Keklik et al. [17] demonstrated that pulsed UV irradiation did not significantly affect HU values, which supports the finding in this study that UV treatment does not negatively impact albumen quality.

The degradation of albumen during storage is largely attributed to the loss of carbon dioxide, which alters the bicarbonate buffering system and causes an increase in albumen pH [34,46]. In our study, albumen pH increased significantly with both storage time and temperature (*p* < 0.05) (Figure 5b). Elevated pH results in depolymerization of ovomucin–lysozyme complexes, leading to thinning of the thick albumen and reduction in HU values.

Overall, storage conditions, particularly temperature, had a greater influence on albumen quality than the sanitization treatment. Under refrigerated conditions (7 °C), the HU scores and thick albumen ratios of washed eggs (NC, NUVC) were comparable to those of unwashed eggs (UC), indicating that proper storage can effectively mitigate the potential negative effects of egg washing on internal quality.

### 3.4. Air Cell Size and Yolk Quality

**Figure 6:**
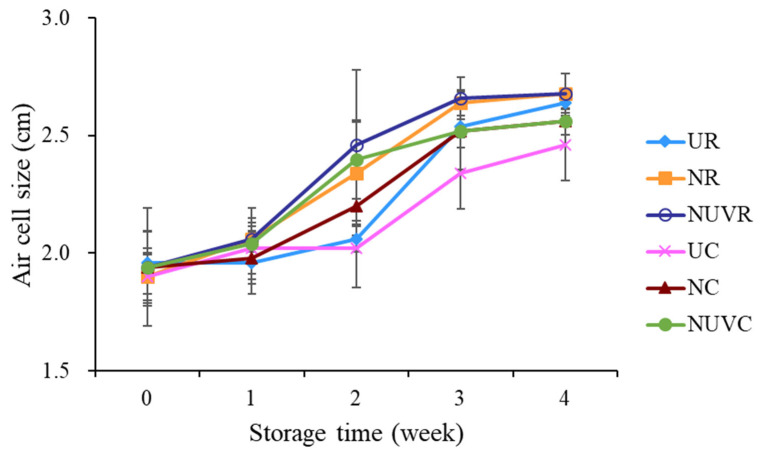
Change in air cell size of eggs with different washing treatments and stored at 7 °C and 25 °C for 4 weeks. UR: unwashed and stored at 25 °C; NR: washed with NaOCl sanitizer and stored at 25 °C: NUVR: washed with NaOCl sanitizer, UV-irradiated, and stored at 25 °C; UC: unwashed and stored at 7°C; NC: washed with NaOCl sanitizer and stored at 7 °C; NUVC: washed with NaOCl sanitizer, UV-irradiated, and stored at 7 °C.

As shown in Figure 6, air cell size increased significantly with prolonged storage duration and elevated storage temperature (*p* < 0.05). This phenomenon can be attributed to the evaporation of water and carbon dioxide through the gas-exchange pores of the eggshell [6]. After four weeks of storage, eggs in the UC group exhibited the least change in air cell size compared to NC and NUVC groups, indicating better preservation under low-temperature conditions.

**Figure 7:**
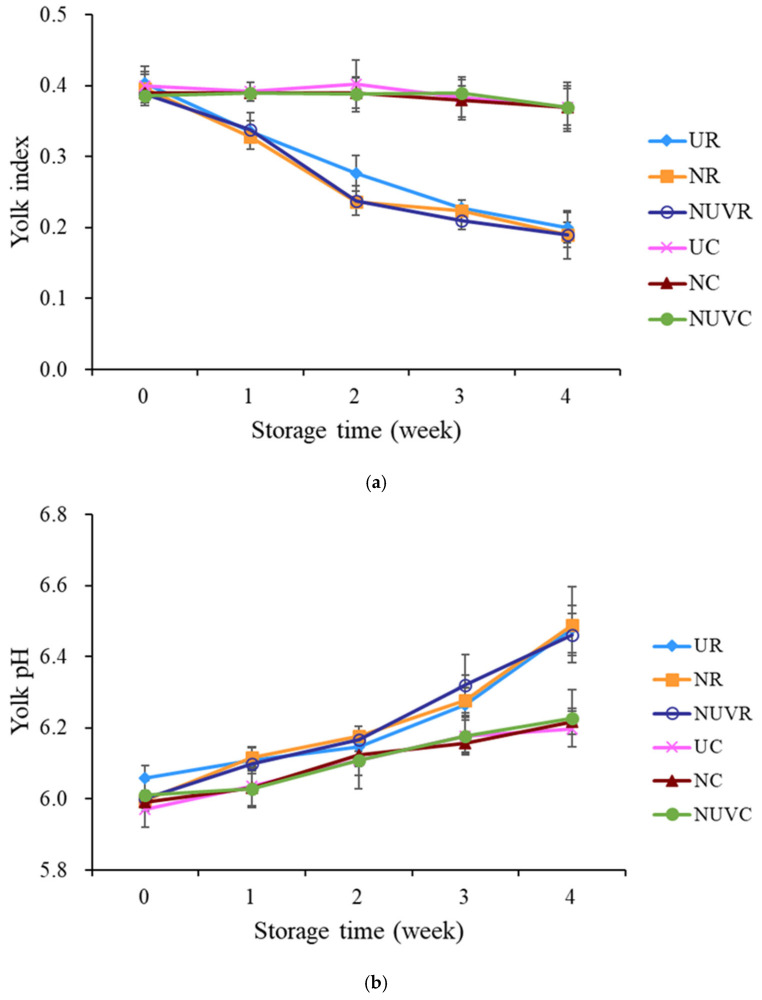

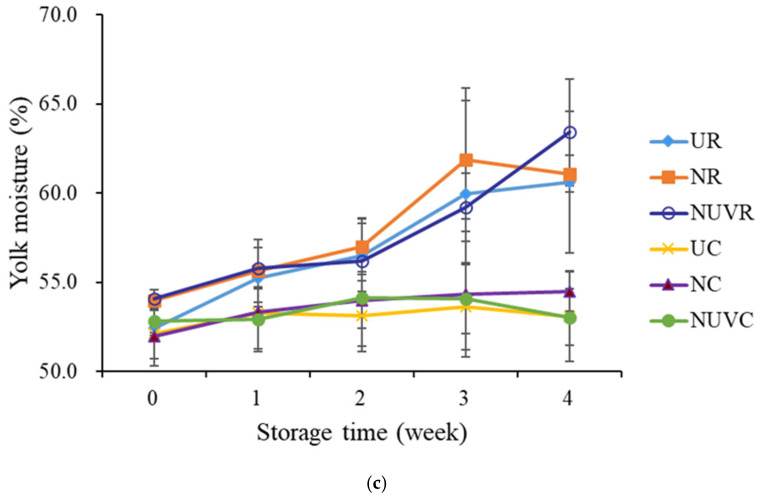
Change in (**a**) yolk index, (**b**) yolk pH value, and (**c**) yolk moisture content of eggs with different treatments and stored at 7 °C and 25 °C for 4 weeks. UR: unwashed and stored at 25 °C; NR: washed with NaOCl sanitizer and stored at 25 °C: NUVR: washed with NaOCl sanitizer, UV-irradiated, and stored at 25 °C; UC: unwashed and stored at 7 °C; NC: washed with NaOCl sanitizer and stored at 7 °C; NUVC: washed with NaOCl sanitizer, UV-irradiated, and stored at 7 °C.

Egg yolk quality was evaluated using the yolk index (YI), defined as the ratio of yolk height to yolk diameter on a flat surface [13]. As depicted in Figure 7a, YI values declined significantly in eggs stored at 25 °C (UR, NR, NUVR) after two weeks, while those stored at 7 °C (UC, NC, NUVC) retained significantly higher YI values throughout the 4-week period (*p* < 0.05). This degradation is primarily due to the weakening of the vitelline membrane as eggs age, allowing water to migrate from the albumen into the yolk. This results in yolk flattening, structural collapse, and a decrease in yolk index. In contrast, refrigeration slows membrane degradation and water transfer, thereby preserving yolk sphericity and quality [13,46].

Yolk pH increased progressively with storage time and temperature, as shown in Figure 7b, largely due to the diffusion of CO_2_ from the egg interior, similar to the pattern observed in the albumen [46]. Interestingly, changes in yolk moisture content (Figure 7c) exhibited a reverse trend compared to that of the albumen (Figure 5c). While albumen moisture decreased over time, yolk moisture content increased, consistent with the hypothesis of water migration from the albumen to the yolk. This moisture transfer contributes to the dilution of yolk solids and membrane weakening, both of which compromise yolk quality. Notably, low-temperature storage effectively suppressed this water migration, further emphasizing the importance of refrigeration in maintaining egg internal quality during prolonged storage [13,46].

## 4. Conclusions

This study demonstrated that integrating UV-C irradiation following NaOCl sanitization effectively reduces the bacterial load on eggshell surfaces without compromising internal egg quality during storage. Among all treatment groups, eggs subjected to the combined NaOCl + UV-C treatment under refrigeration (NUVC) maintained comparable albumen and yolk quality to those in the unwashed, refrigerated control group (UC) throughout the 4-week storage period.

Storage temperature and duration were found to be the primary factors influencing internal egg quality, with higher temperatures and longer storage times significantly reducing albumen height, Haugh unit, and yolk index. Meanwhile, ATR–FTIR analysis revealed intensified OH absorption peaks under cold storage, suggesting cuticle dehydration and the potential development of surface fissures, which may alter shell permeability.

These findings support the combined NaOCl and UV-C sanitization strategy as a safe and effective approach to enhance microbial safety while preserving the functional and structural integrity of eggs, particularly under the warm, humid conditions typical of subtropical environments. The use of UV-C as a post-washing treatment can potentially offer an energy-efficient, chemical-minimizing solution for the egg processing industry.

However, this was limited to evaluating total aerobic bacterial counts as the primary microbial indicator. Future studies should incorporate a broader spectrum of pathogenic and spoilage organisms, including *Salmonella* spp., coliforms, and psychrotrophic bacteria, to comprehensively assess microbial risk.

Emerging sustainable disinfection technologies—including pulsed UV, non-thermal atmospheric plasma, and electrolyzed water—warrant further exploration. Future research should focus on optimizing treatment conditions (e.g., dose, duration, and surface contact), evaluating long-term effects on eggshell integrity and internal quality, and conducting cost-benefit analyses for commercial implementation. Additionally, the development of user-friendly, energy-efficient equipment adapted to various production scales can improve industry adoption. These efforts can support the establishment of standardized, eco-friendly egg disinfection systems aligned with food safety and sustainability goals.

## Figures and Tables

**Table 1:** Changes in total aerobic bacterial counts on eggshell surfaces and in egg contents under different treatments during 4-week storage period at 7 °C and 25 °C.

Treatment *	Eggshell ** (log CFU/mL)	Egg Content ** (log CFU/g)				
	Week 0	Week 0	Week 1	Week 2	Week 3	Week 4
Unwashed, 25 °C (UR)	4.48 ± 0.24 ^a^	<1	<1	<1	0	0
NaOCl-washed, 25 °C (NR)	3.25 ± 0.26 ^b^	<1	0	0	<1	<1
NaOCl + UV, 25 °C (NUVR)	3.00 ± 0.22 ^c^	<1	0	0	0	<1
Unwashed, 7 °C (UC)	-	<1	<1	0	0	<1
NaOCl-washed, 7 °C (NC)	-	<1	0	<1	0	0
NaOCl + UV, 7 °C (NUVC)	-	<1	<1	<1	0	0

^a–c^ Means in the same row with different superscripts differ significantly (*p* < 0.05). * U: unwashed; N: washed with NaOCl sanitizer; NUV: washed with NaOCl sanitizer and UV-irradiated; UR: unwashed and stored at 25 °C; NR: washed with NaOCl sanitizer and stored at 25 °C: NUVR: washed with NaOCl sanitizer, UV-irradiated, and stored at 25 °C; UC: unwashed and stored at 7 °C; NC: washed with NaOCl sanitizer, and stored at 7 °C; NUVC: washed with NaOCl sanitizer, UV-irradiated, and stored at 7 °C. ** Mean ± S.D. (*n* = 12 for eggshell; *n* = 3 for egg content). “<1” indicates bacterial counts were below detection limit (i.e., <10 CFU/g); “0” indicates no colony growth was observed on the plate.

## Data Availability

The original contributions presented in the study are included in the article. Further inquiries can be directed to the corresponding author.
